# Base water potential but not hydrotime predicts seedling emergence of *Medicago sativa* under water stress conditions

**DOI:** 10.7717/peerj.13206

**Published:** 2022-05-10

**Authors:** Xianglai Chen, Zhichao Wei, Dali Chen, Xiaowen Hu

**Affiliations:** State Key Laboratory of Grassland Agro-Ecosystems; Key Laboratory of Grassland Livestock Industry Innovation, Ministry of Agriculture and Rural Affairs; Engineering Research Center of Grassland Industry, Ministry of Education; College of Pastoral Agricult, Lanzhou, China

**Keywords:** Drought, Hydrotime model, *Medicago sativa*, Seedling emergence, Base water potential

## Abstract

We determined the hydrotime model parameters of 10 alfalfa seed lots by incubating seeds at various water potentials in the laboratory. Meanwhile, seedling emergence under drought and salinity conditions in a greenhouse pot experiment, and seedling establishment in the field were determined. Correlation analysis was utilized to detect the relationship between hydrotime model parameters and seedling emergence under water stress conditions. The germination percentage did not differ significantly among seed lots when seeds were incubated at −0.1 MPa, while it differed significantly among seed lots at water potentials of −0.3 and −0.6 MPa. Compared to control conditions, drought and salinity decreased seedling emergence to different extents, depending on seed lots. Seedling emergence in the field differed significantly among seed lots and ranged from 30% to 80%. *Ψ*_b(50)_ showed a significant correlation with seedling emergence under various conditions and with seedling establishment in the field, while no correlation was observed between *θ*_H_, *σ*_φb_ and seedling emergence and establishment. These results suggest that *Ψ*_b(50)_ can be used to rank the vigor of alfalfa seed lots and thus predict seedling emergence and establishment under water stress conditions.

## Introduction

Alfalfa (*Medicago sativa* L.), an important perennial legume forage crop with high nutritional content, is one of the most important crops in China and is widely cultivated in arid and semi-arid regions (Kong et al., 2020; Su, 2007). However, poor establishment of this species has become one of the major constraints encountered by farmers (Guo et al., 2005), due to local harsh environments, *e.g.*, water scarcity, salinization and soil erosion (Wang et al., 2004b). Thus, improving seed germination and seedling establishment under stressful conditions have become a key determinant of *M*. *sativa* cultivation and utilization.

Selection for high vigor alfalfa seeds is an effective way to enhance seedling establishment and consequently hay yield in the field. Previous studies (Xia et al., 2020; Yacoubi et al., 2013) showed that there was an advantage in using high vigor seeds to establish alfalfa fields because low vigor seeds resulted in limited seedling emergence. Wang et al. (2004a) found a positive relationship between seed vigor and seedling emergence in the field. Selection for high vigor seeds also has proven to be beneficial for seedling emergence and yield of other crop species, *e.g.*, maize, sorghum (Kim et al., 2013; Mondo et al., 2013).

There are many ways to evaluate seed vigor, such as seedling growth dynamics (Tohidloo & Kruse, 2009; Sharma et al., 2014; Wang, Liu & Song, 2015), electrical conductivity (EC) (Hampton, Leeks & McKenzie, 2009; Matthews et al., 2009; Sibelle et al., 2012; Jonathan et al., 2013), accelerated aging (Mcdonald & Phaneendranath, 1978; Souza et al., 2017), radicle emergence (Lv, Wang & Powell, 2016; Guan et al., 2018). Wang et al. (2004a) showed that EC and accelerating aging (AA) are more sensitive for evaluating alfalfa seed vigor than the standard germination test. Pan et al. (2017) showed that the standard germination test failed to detect differences in seed vigor, while EC and radicle emergence were highly sensitive ways to determine seed vigor.

Many studies have found that the germination rate (*GR*g, the reciprocal of time to a given germination fraction, 1/*t*g) is linearly related to water potential (Gummerson, 1986; Bradford, 1990; Dahal & Bradford, 1994). Thus, the hydrotime model has been developed to evaluate the effect of water potential on progress towards germination (Gummerson, 1986; Bradford, 2002; Hu et al., 2015). The hydrotime modeling approach can help predict seed germination under water deficit conditions, as reported by Patanè and Tringali for seeds of *Brassica carinata* (Patanè & Tringali, 2011). In particularly, the base or threshold water potential for a specific germination fraction has been reported to be closely related to seed vigor in many species, *e.g.*, sugar beet, cotton, and rapeseed (Farzane & Soltani, 2011; Soltani & Farzaneh, 2014; Soltani et al., 2017). Castillo-Lorenzo et al. (2019) indicated that 10 *Brassica* seed lots had diverse seed germination phenotypes, with hydrotimes (*θ*_H_) differing by 3 to 7-fold and base water potentials (*Ψ*_b_) by −1.5 MPa, illustrating hydrotime parameters can be used to explain seed vigor (Castillo-Lorenzo et al., 2019). Li et al. (2017) found that priming improved the *Ψ*_b(50)_ for alfalfa seeds and increase germination speed and seedling growth under high salinity conditions. This study implied that a change in hydrotime model parameters induced by priming may be closely related to performance of seeds and seedlings under the stressful field conditions. However, the assumption that hydrotime model parameters can be used to predict seed vigor and their performance under various stressful conditions has not been tested. In our study, we asked the following questions. (1) Does the hydrotime model predict seed germination in response to water stress for *M*. *sativa* seed lots with various levels of vigor? (2) Are there any correlations between hydrotime parameters and seedling emergence under stressful conditions?

## Materials and Methods

### Germination test

Ten seed lots of alfalfa from different company and production sites were provided by the Forage and Turfgrass Seed Quality Inspection Test Center, Lanzhou, Ministry of Agriculture and Rural Affairs, China. According to a preliminary study, these ten seed lots have similar germination percentage under standard germination test condition at 20 °C (ISTA, 2014).

A germination test for each seed lot was conducted by incubating seeds at 20 °C in 12/12 h light/dark at water potentials of −0.1, −0.3, −0.6 and −0.9 MPa. Polyethylene glycol 6000 (PEG) solutions were prepared according to Michel & Kaufmann (1973). In addition, as described in Hu et al. (2015), the water potential of solutions was calibrated using a Dew Point Microvoltmeter HR-33T (Wescor, Logan, UT, USA). For each treatment, three replicates of 50 seeds each were placed in 11 cm diameter Petri dishes on two sheets of filter paper moistened with 7 mL of PEG solution, then the Petri dishes were enclosed with parafilm to prevent evaporation of water. Seeds were transferred to new filter paper with new solution every 3 d to ensure relatively constant water potential in the treatments (Hu et al., 2015; Zhang et al., 2020). Seeds were counted for germination every 6, 18 and 24 h, depending on germination speed, for 20 d and seedlings removed at each counting. Seeds were considered to be germinated when the radicle had emerged to at least half the seed length. Final germination percentage was expressed as germinated seeds divided by total sown seeds. The germination rate was expressed as 1/t_50_. The t_50_ was the time to 50% of the maximum germination percentage in the most favorable environment for each alfalfa seedlot, it was estimated from the function according to Steinmaus, Prather & Holt (2000).

### Greenhouse experiment

The pot experiment was conducted in the greenhouse at Yuzhong Campus of the Lanzhou University (35°85′N,104°12′E, 1,720 m a.s.l.), Gansu Province, China. Mean daily temperature is 20 °C (13–27 °C) and verage air humidity is about 60% (30–90%). In addition, average photosynthetic photon flux densities (PPFD, 48.4-85.3 k lx) is 50–80% natural light. Seeds of the 10 alfalfa lots were sown in soil at control (90% relative field capacity), water stress (60% relative field capacity) and salinity stress (NaCl and soil were uniformly mixed at 1:1,000). Field capacity of the soil was determined according to Hu et al. (2018). Field capacity of soil was 21.21%. For each treatment, three replicates of 50 seeds each seed lot were sown pots (20 cm tall, 15 cm in diameter) that contained 3.00 kg soil dried at 105 °C. Based on the Chinese Soil Classification System (Gong, 1999), the soil used in this study was a cultivated loess soil. According to soil moisture characteristic curves from previous study (Ma et al., 2005), the soil water potential for 90% and 60% of field capacity is about −0.1 MPa and −0.6 MPa at 20 °C. No fertilizer was applied during the whole experimental period. To prevent water from escaping from the bottom of the pot, plastic bag was put inside the pot and a PVC tube was inserted into pot. Pots were observed daily and soil moisture content was controlled by weighing the pot and adding more water if needed until complete seedling emergence. The experiment applied completely random design. Final emergence percentage (EP) and emergence index (EI) were determined as follows:

(1) $${\rm EP}= (n/N))\times {100}{\%}$$

(2) $${\rm EI} = \sum {(E_{\rm t} /D_{\rm t}) }$$

where *n* is the number of emerged seeds, *N* was the total number of tested seeds; *D*_t_ is the days since sowing, *E*_t_ is the number of emerged seeds that day corresponding to *D*_t_.

### Field experiment

The field experiment was carried out at the Yuzhong Campus, where mean annual precipitation and mean annual temperature are 350 mm and 6.7 °C, respectively. Four replicates of 200 seeds for each seed lot were sown in a block by drilling method in April 2018. The experiment applied completely random design. Seedlings were counted for field emergence every two days for two months, when final emergence percentage and emergence index (as indicated in Eq. (1)) were determined. During the whole experimental period, the temperature is typically ranged from 5–22 °C, and total rainfall is about 112 mm.

### Data analysis

The effect of seed lot and water potential or environment conditions (the control, water stress and salt stress) on seed germination percentage, germination rate (1/t_50_), seedling emergence percentage and emergence index were tested by fitting generalized linear mixed models (GLMMs). Seed lot and water potential or environment conditions were included as fixed effects and replicates as a random effect. Seed germination percentage and seedling emergence percentage was a probability ranging from 0 to 1, hence, for the GLMMs of these two parameters, a binomial estimation of the model with a logit link function were applied. Duncan’s test was used to compare means when significant differences were found. Pearson correlation analysis was used to detect the relationship between *θ*_H_, *Ψ*_b(50)_, *σ*_*Ψ*b_ and germination, germination rate, emergence percentage, emergence index.

The hydrotime constant *θ*_H_ (MPa·h) and base water potential *Ψ*_b_(g) (MPa) for a specific germination fraction (g) were calculated using the hydrotime model (Gummerson, 1986; Bradford, 1990), More details about hydrotime model see supporting information.

(3) $${\theta _{\rm{H}}} = [\Psi  - {\Psi _b}({\rm{g}})]{t_g}$$

(4) $${\rm{probit}}({\rm{g}}) = [\Psi  - ({\theta _{\rm{H}}}/{t_{\rm{g}}}) - {\Psi _{b(50)}}]/{\sigma _{\Psi b}}$$

## Results

### Effect of water potential on seed germination

Seed lot (SL), water potential (WP) and their interactions had significant effects on germination percentage (GP) (Table 1). Germination percentage differed significantly across seed lots, except −0.1 MPa. With decreasing WP, GP decreased to some extent depending on the seed lot. For example, GP of seeds lots 3 and 6 decreased from 89% to 3% and from 91% to 33%, respectively, when WP decreased from −0.1 to −0.9 MPa.

**Table 1:** Seed germination percentage of 10 seed lots of alfalfa in response to water potential.

Seed lot	Water potential (MPa)			
	−0.1	−0.3	−0.6	−0.9
1	93.96 ± 0.04 Aabcd	94.58 ± 0.71 Aab	84.32 ± 1.68 Ba	12.00 ± 3.06 Cbcd
2	87.48 ± 1.27 Ae	79.22 ± 2.82 Ad	34.67 ± 4.67 Bc	6.08 ± 0.08 Ccd
3	89.33 ± 4.06 Ade	86.67 ± 1.33 Ac	26.81 ± 1.90 Bc	3.33 ± 2.40 Cd
4	98.00 ± 1.15 Aa	94.61 ± 2.39 Aab	83.49 ± 2.26 Aa	16.88 ± 7.91 Bbc
5	97.33 ± 1.33 Aab	95.28 ± 1.82 Aab	56.39 ± 15.81 Bb	10.00 ± 3.06 Cbcd
6	91.33 ± 1.76 Acde	92.00 ± 1.15 Aabc	86.67 ± 1.76 Aa	32.88 ± 2.89 Ba
7	95.28 ± 1.82 Aabc	98.00 ± 2.00 Aa	55.36 ± 6.03 Bb	18.84 ± 4.81 Cb
8	91.93 ± 1.21 Abcde	90.00 ± 3.06 Abc	53.70 ± 0.91 Bb	8.75 ± 2.88 Cbcd
9	93.33 ± 1.33 Aabcd	91.28 ± 1.75 Aabc	60.67 ± 5.93 Bb	6.00 ± 2.31 Ccd
10	97.33 ± 0.67 Aab	94.49 ± 3.02 Aab	62.49 ± 5.76 Bb	11.33 ± 2.67 Cbcd

Source of variation	DF	Germination (%)	
		Chisq	*P*
Seed lot (*SL*)	9	22.870	0.006
Water potential (*WP*)	3	202.210	<0.001
*SL* × *WP*	27	96.161	<0.001

**Note:**

Different uppercase indicated significant difference among different water potential for given seed lot at the 0.05 level (*P* < 0.05), different lowercase indicated significant difference among different seed lots for given water potential at the 0.05 level (*P* < 0.05). The effect of seed lot and water potential on seed germination were analyzed using generalized linear mixed models. Three replications for each treatment of each seed lot. Duncan’s test was used to compare means when significant differences were found.

**Figure 1:**
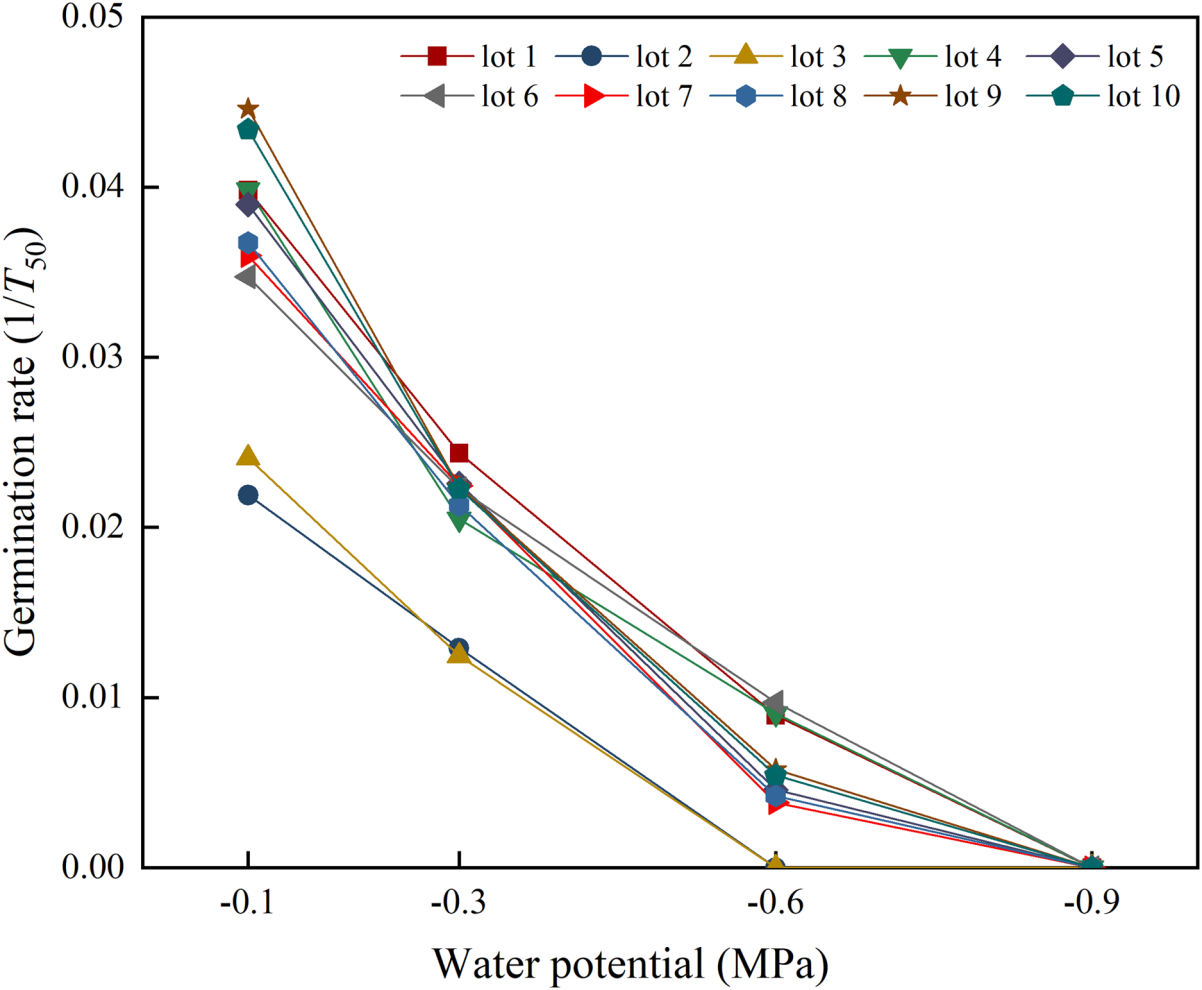
Seed germination rate (1/t_50_) of 10 seed lots of alfalfa in response to water potentials. Three replications for each treatment of each seed lot.

Seed lot, water potential and their interactions had significant effects on germination rate (Fig. 1). Germination rate differed across seed lots at all water potentials, except −0.9 MPa. Germination rate decreased in response to decreasing water potential, and no seed lots germinated to 50% when sown at −0.9 MPa.

### Hydrotime analysis for seed germination in response to water potential

**Figure 2:**
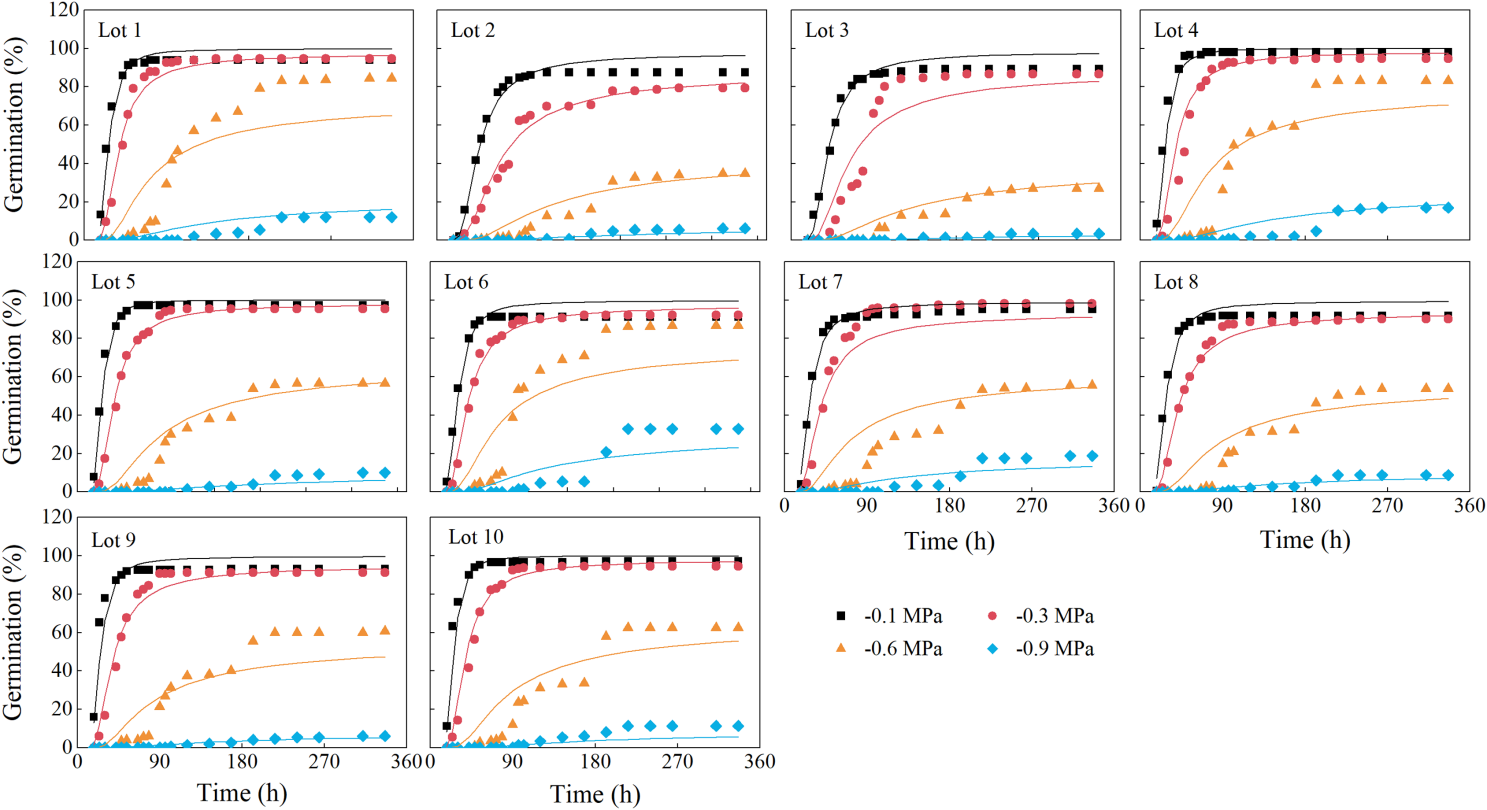
Germination time courses of 10 alfalfa seed lots. The symbols are the observed data and the curves are the results of fitting by the hydrotime model. Three replications for each treatment of each seed lot.

The predicted germination time courses at the four water potentials generally fit the observed germination data very well, with *R*^2^ values of 0.82–0.97 (Fig. 2; Table 2). Estimated values of hydrotime (*θ*_H_), base water potential [*Ψ*_b(50)_] and *σ*_*φ*b_ differed among seed lots (Table 2). The value of *θ*_H_ ranged from 13.37 to 22.23 MPa·h, the *Ψ*_b(50)_ varied from −0.8 to −0.6 MPa, and *σ*_*φ*b_ varied from 0.17 to 0.25.

**Table 2:** Estimated hydrotime model parameters for 10 alfalfa seed lots.

Seed lot	*θ*_H_ (MPa·h)	*Ψ*_b(50)_ (MPa)	*σ*_*φ*b_	*R* ^2^
1	17.00	−0.74	0.22	0.82
2	22.23	−0.57	0.23	0.97
3	18.65	−0.55	0.20	0.93
4	18.32	−0.77	0.21	0.89
5	15.54	−0.68	0.17	0.94
6	20.56	−0.78	0.25	0.85
7	16.18	−0.68	0.24	0.90
8	15.26	−0.64	0.21	0.95
9	13.37	−0.63	0.19	0.89
10	14.60	−0.67	0.17	0.91

**Note:**

The *θ*_H_ is constant hydrotime, *Ψ*_b(50)_ is base water potential for 50% of seeds to germinate, *σ*_*φ*b_ is standard deviation of *Ψ*_b(50)_. Coefficients determination (*R*^2^) is showing the goodness of fitness of the model fitting. Three replications for each treatment of each seed lot.

### Effect of water and salinity stress on seedling emergence in the greenhouse

Seed lot, environmental condition and their interactions had significant effects on seedling emergence percentage (EP) in the greenhouse (Table 3). Emergence percentage decreased with water and salinity stress compared to the control and differed significantly across the seed lots at each environmental condition. For example, seedling emergence in the control, water stress and salinity stress was 55%, 23% and 17%, respectively, for lot 3, while it was 81%, 46% and 29%, respectively, for lot 8. Seed lot and environment condition had significant effects on emergence index, however, there was no significant effect for their interactions (Table 3).

**Table 3:** Effect of environmental conditions on seedling emergence percentage (EP) and emergence index (EI) of 10 alfalfa seed lots.

Seed lot	Control		Water stress		Salinity stress	
	EP (%)	EI	EP (%)	EI	EP (%)	EI
1	93.33 ± 2.40 Aa	7.15 ± 0.85 Aa	42.00 ± 2.00 Bab	1.64 ± 0.20 Ba	41.33 ± 4.67 Ba	1.38 ± 0.17 Ba
2	74.67 ± 7.68 Ab	4.25 ± 0.70 Abc	36.67 ± 0.67 Bb	1.36 ± 0.11 Ba	27.33 ± 2.67 Bb	0.87 ± 0.07 Bcd
3	54.67 ± 2.67 Ac	2.73 ± 0.46 Ac	23.33 ± 2.40 Bc	0.78 ± 0.06 Bb	16.67 ± 1.76 Bc	0.48 ± 0.03 Bd
4	90.00 ± 5.29 Aab	5.66 ± 0.74 Aab	42.67 ± 3.53 Bab	1.85 ± 0.31 Ba	36.00 ± 3.06 Bab	1.34 ± 0.19 Bab
5	85.33 ± 5.46 Aab	5.25 ± 0.39 Aab	46.00 ± 3.06 Ba	1.86 ± 0.13 Ba	32.00 ± 1.16 Cab	1.12 ± 0.02 Babc
6	90.67 ± 3.33Aa	6.49 ± 0.86 Aa	42.67 ± 2.67 Bab	1.90 ± 0.20 Ba	34.67 ± 3.53 Bab	1.40 ± 0.16 Ba
7	87.33 ± 1.76 Aab	5.57 ± 0.23 Aab	38.67 ± 2.91 Bab	1.48 ± 0.01 Ba	37.33 ± 2.40 Bab	1.30 ± 0.11 Bab
8	81.33 ± 7.33 Aab	4.02 ± 0.50 Abc	46.00 ± 4.00 Ba	1.63 ± 0.25 Ba	29.33 ± 4.06 Bb	0.93 ± 0.07 Bbc
9	88.00 ± 4.00 Aab	5.44 ± 0.42 Aab	40.67 ± 2.91 Bab	1.54 ± 0.07 Ba	28.00 ± 2.31 Cb	0.94 ± 0.12 Bbc
10	88.67 ± 3.33 Aab	5.70 ± 0.62 Aab	43.33 ± 1.33 Bab	1.69 ± 0.11 Ba	32.67 ± 3.71 Bab	1.15 ± 0.21 Babc
Source of variation		DF	EP		EI	
			Chisq	*P*	Chisq	*P*
Seed lot (*SL*)		9	109.933	<0.001	137.120	<0.001
Environmental conditions (*EC*)		2	73.432	<0.001	182.878	<0.001
*SL × EC*		18	36.683	0.006	64.685	<0.001

**Note:**

Different uppercase indicated significant difference among different stress treatments for given seed lot at the 0.05 level (*P* < 0.05), different lowercase indicated significant difference among different seed lots for given stress treatment at the 0.05 level (*P* < 0.05). The effect of seed lot and environmental conditions on seedling emergence percentage and emergence index were analyzed using generalized linear mixed models. Three replications for each treatment of each seed lot. Duncan’s test was used to compare means when significant differences were found.

### Effect of field sowing on seedling emergence

**Figure 3:**
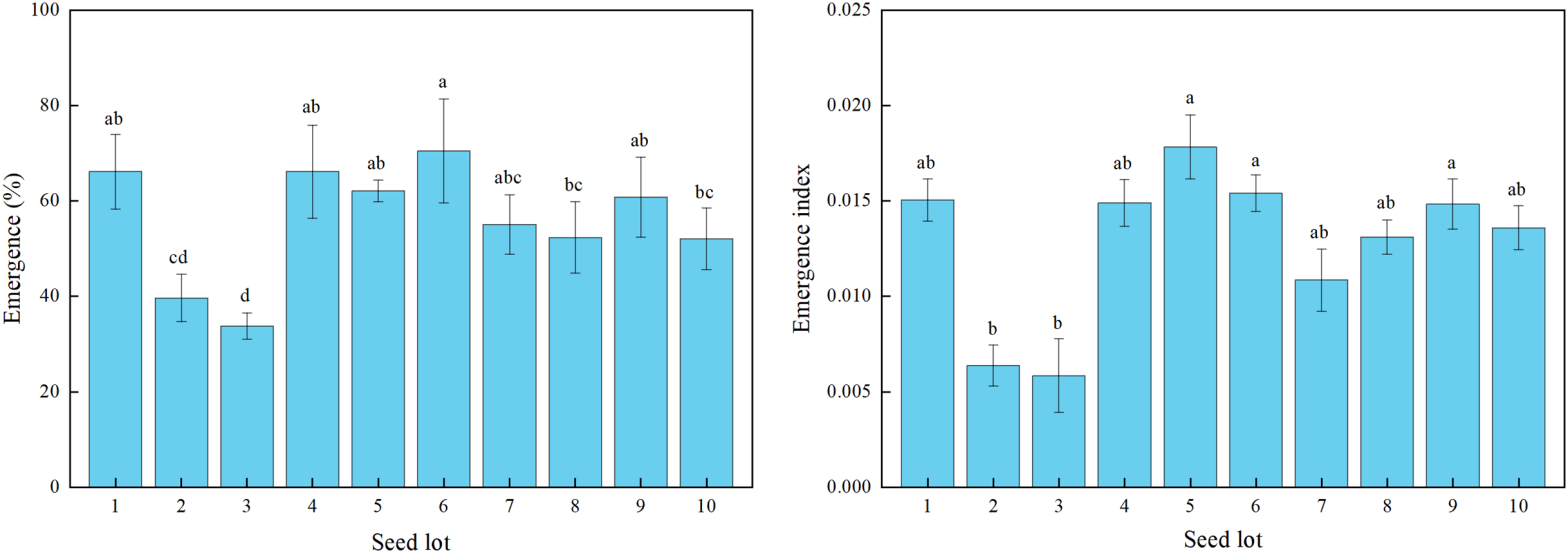
Seedling emergence percentage and emergence index for 10 alfalfa seed lots under field sowing. Different lowercase letters indicated significant difference among different seed lots at the 0.05 level (*P* ≤ 0.05). Three replications for each treatment of each seed lot. Duncan’s test was used to compare means when significant differences were found.

There were significant differences among the 10 alfalfa seed lots sown in the field for seedling emergence percentage and index (Fig. 3). The seedling emergence percentage ranged from 34% for lot 3 to 71% for lot 6.

### Correlation between hydrotime parameters, seed germination and seedling emergence

The results indicated a correlation between traits of germination, emergence and hydrotime model parameters (Table 4). There was a negative significant relationship between base water potential [*Ψ*_b(50)_] and germination percentage (r = −0.983, *P* < 0.001), germination rate (r = −0.746, *P* = 0.013), emergence percentage (r = −0.807, *P* = 0.005; r = −0.624, *P* = 0.054; r = −0.834, *P* = 0.003), emergence index (r = −0.841, *P* = 0.002; r = −0.813, *P* = 0.004; r = −0.927, *P* < 0.001) under control condition, water stress and salinity stress, respectively, and between base water potential [*Ψ*_b(50)_], field emergence percentage (r = −0.751, *P* = 0.012) and index (r = −0.728, *P* = 0.017). On the other hand, there was no relationship between *θ*_H_, *σ*_ψb_ with germination, emergence traits.

**Table 4:** Pearson correlation coefficient between overall mean of seed germination percentage and rate (mean of four levels of water potential and three replicates), emergence percentage and index and hydrotime parameters (*θ*_H_, *Ψ*_b(50)_, *σ*_φb_) at different environmental conditions of control, water stress, salinity stress and field sowing for 10 alfalfa seed lots.

Variable		*θ*_H_ (MPa•h)	*Ψ*_b(50)_ (MPa)	*σ*_*φ*b_
Laboratory	Germination (%)	−0.152	−0.983 (<0.001)	0.206
	Germination rate (h^−1^)	-0.641 (0.046)	−0.746 (0.013)	−0.204
Greenhouse	Control condition			
	Emergence (%)	−0.317	−0.807 (0.005)	0.086
	Emergence index	−0.136	−0.841 (0.002)	0.196
	Water stress			
	Emergence (%)	−0.368	−0.624 (0.054)	−0.120
	Emergence index	−0.185	−0.813 (0.004)	−0.011
	Salinity stress			
	Emergence (%)	−0.124	−0.834 (0.003)	0.280
	Emergence index	−0.039	−0.927 (<0.001)	0.320
Field	Field experiment			
	Emergence (%)	−0.457	−0.751 (0.012)	−0.043
	Emergence index	−0.424	−0.728 (0.017)	0.129

**Note:**

The *θ*_H_ is constant hydrotime, *Ψ*_b(50)_ is base water potential for 50% of seeds to germinate, *σ*_*φ*b_ is standard deviation of *Ψ*_b(50)_. Numbers in parentheses indicate the significance of the coefficients. Three replications for each treatment of each seed lot.

## Discussion

Drought and salinity are the main factors affecting seedling establishment as well as crop production across the world (McMillan, 1971; Isselstein, Tallowin & Smith, 2002; Gu et al., 2018). Our study clearly showed that drought stress significantly decreased seed germination in the laboratory and seedling emergence in the greenhouse. Moreover, seedling emergence in the field was far less than the potential germination capacity of the seeds, suggesting that harsh environmental conditions play a key role in determining seedling establishment and consequently production of alfalfa hay on the Loess Plateau (Zhao, Liu & Li-Jie, 2005; Yang & Yong, 2010; Li, Zhang & Sun, 2015). However, the detrimental effect of stress can be alleviated by high quality seeds, *e.g.*, seedling emergence of 10 lots ranged from 30–70% in the field, although all seed lots germinated to higher than 87% in the laboratory. Further, germination performance under moderately stressful conditions in the lab failed to detect seed quality difference among seed lots, and thus germination percentage was not a good indicator of seed vigor in our study. This result is consistent with those of Pan et al. (2017) and Wang et al. (2003) who found that standard germination tests are poor in ranking seed quality, and they show no significant correlation with seed/seedling performance in the field. Interestingly, the germination percentage differed more as the water potential decreased from −0.1 to −0.6 MPa, suggesting germination at appropriate stressful conditions is helpful in distinguishing seed quality of alfalfa. In our study, −0.6 MPa was an ideal germination condition for seed quality evaluation, since the highest variation among seeds lots was observed under this level of stress.

Hydrotime models are used to describe the dynamics of seed germination in response to reduced water availability (Windauer, Altuna & Benech-Arnold, 2007; Cardoso & Bianconi, 2013), and the model allowed a better description of the seed germination time courses at reduced osmotic potential in carob and mangroves (Cavallaro et al., 2016; Torabi et al., 2016; Farahinia et al., 2017; Wijayasinghe et al., 2016). In agreement with these studies, our study clearly showed that hydrotime model also could describe the germination time course of different alfalfa seed lots at various water potentials very well, with R^2^ values of 0.82–0.97 (Fig. 2; Table 2). A distinct advantage of the hydrotime model for seeds incubated across a range of water potentials is that variation or similarity in germination among seed lots can be ascribed to specific underlying factors such as *θ*_H_, *Ψ*_b(50)_, and *σ*_*φ*b_ (Allen et al., 2000). Dahal & Bradford (1990) showed that seeds with lower *Ψ*_b_(g) and *θ*_H_ values generally had higher vigor than those with high values, which allowed seeds to germinate rapidly under stressful condition. Consistent with this, seed lot 6 with the lowest value of *Ψ*_b(50)_ had the highest seedling establishment, while seed lot 3 with the highest *Ψ*_b(50)_ had the lowest seedling establishment in the field. Moreover, our study showed significant correlations between *Ψ*_b(50)_ and seed germination and seedling emergence under water stress conditions, further supporting the assumption that tolerance of alfalfa seed lots to stressful conditions can be identified by a decrease in *Ψ*_b(50)_. Thus, *Ψ*_b(50)_ can be applicable for predicting early seed vigor and seedling establishment in the field.

On the other hand, no correlation between *θ*_H_ or *σ*_φb_ and seedling emergence was observed in our study. A possible reason is that a lower *θ*_H_ favors rapid and uniform germination; however, this occurs only when seeds are subjected to moist conditions. According to the hydrotime definition, when external water potential approaches or is lower than *Ψ*_b(50)_, seeds will accumulate the hydrotime units very slowly, which overrides the advantage of low *θ*_H_. On the Loess Plateau, seedling emergence after sowing is completely dependent on the unpredictable rainfall, and drought frequently occurs even during the rainy season. This unpredictable drought stress may partly explain why *Ψ*_b(50)_ rather than *θ*_H_ contribute to the difference in seedling establishment among seed lots. Moreover, no correlation between *θ*_H_ and *Ψ*_b(50)_ was observed in our study, suggesting that the role of these two parameters may act independently in regulating seed germination and seedling emergence in the field. It seems reasonable to hypothesis that seeds with low *θ*_H_ will have an advantage in moist conditions, while seeds with low *Ψ*_b(50)_ will have an advantage during drought stress.

## Conclusion

In brief, our study clearly showed that hydrotime model can describe the germination time course of alfalfa seeds in response to various water potentials very well. *Ψ*_b(50)_ can be used to rank alfalfa seed vigor and thus predict seedling emergence and speed under water stress conditions. However, it is also worth noting that current study only test seedling emergence in the field once, and a replicate experiment across sites and years is needed to confirm this conclusion.

## Supplemental Information

10.7717/peerj.13206/supp-1 Supplemental Information 1 Raw data.

10.7717/peerj.13206/supp-2 Supplemental Information 2 Development and calculation of the hydrotime model.

## Additional Information and Declarations

### Data Availability

The following information was supplied regarding data availability: The raw measurements are available in the Supplemental File.
