# Supplementary material for: Base water potential but not hydrotime predicts seedling emergence of Medicago sativa under water stress conditions
Source: PeerJ. 2022 May 10;10:e13206. doi: 10.7717/peerj.13206 (PMC9104092; doi:10.7717/peerj.13206)
Supplement: Supplemental Information 2 [file peerj-10-13206-s002.docx]

Seed germination is a pivotal stage in the life cycle of plants (Walck et al., 2011). Water potential is a critical environmental factor regulating seed dormancy-break, germination and subsequent seedling establishment (Baskin & Baskin, 2014). Thus, knowledge of seed germination responses to water potential is required not only for understanding the ecological adaptation of species but also for formulating effective strategies for restoration (Fenner et al., 2005).

Many studies have found that the germination rate (GR_g_, the reciprocal of time to a given germination fraction, 1/t_g_) is linearly related to water potential (Gummerson, 1986; Bradford, 1990; Dahal & Bradford, 1994). Thus, the hydrotime model has been developed to evaluate the effect of water potential on progress towards germination (Gummerson, 1986; Bradford, 2002). In this model, several parameters were fitted and used to quantify water potential requirements for seed germination, such as the base water potential [*Ψ*_b(g)_, the base or threshold water potential for a specific germination fraction g] and the hydrotime constant for a given seed population (*θ*_H_) (Hu et al., 2015). Generally, implicit in hydrotime models are the assumptions that the hydrotime constant, *θ*_H_, is constant for all fractions of the seed population and that base water potential, *Ψ*_b(g)_, follows a normal distribution with a mean (*Ψ*_b(g50)_) and standard deviation (*σ_Ψ_*_b_). Thus, the hydrotime model can be defined as (Gummerson, 1986; Bradford, 1990):

*θ*_H_ = [*Ψ* - *Ψ*_b(g)_]*t*_g_ (eq.1)

probit(g) = [*Ψ*– (*θ*_H_/*t*_g_) –*Ψ*_b(50)_] /*σ_Ψ_*_b_ (eq.2)

where *θ*_H_ is the hydrotime constant (MPa·h), *Ψ* is the actual water potential of germination testing, *Ψ*_b_(g) the base water potential, and *t*_g_ the actual time to germination of fraction g. The base water potential [*Ψ*_b(50)_] the median water potential to germination, and *σ_Ψ_*_b_ is the standard deviation of requirements *ψ* among individual seeds in the seed lot. The hydrotime model can be used to fit the data using nonlinear regression in SPSS 25.0 (SPSS Inc., Chicago, Illinois, USA). The model parameters, *θ*_H_, *Ψ*_b(50)_ and *σ_Ψ_*_b_ were estimated by an iterative method until the residual sum of squares (RSS) of the regression was minimized (Ellis *et al*., 1986).

**References**

Baskin CC, Baskin JM. 2014. Seeds: ecology, biogeography, and evolution dormancy and germination (2nd edn). San Diego, CA, Academic Press.

Bradford KJ. 2002. Applications of hydrothermal time to quantifying and modelingseed germination and dormancy. *Weed science* 50:248-260.

Bradford KJ.1990. A water relations analysis of seed germination rates. *Plant Physiology* 94: 840-849.

Dahal P, Bradford KJ. 1994. Hydrothermal time analysis of tomato seed germination at suboptimal temperature and reduced water potential. *Seed Science Research* 4:71-80.

Ellis RH, Covell S, Roberts EH, Summerfield RJ. 1986. The influence of temperature on seed-germination rate in grain legumes. 2. Intraspecific variation in chickpea (*Cicer arietinum* L.) at constant temperatures. *Journal of Experimental Botany* 37: 1503–1515.

Fenner MK, Fenner M, Thompson K. 2005. The ecology of seeds. Cambridge, Cambridge University Press.

Gummerson RJ. 1986. The effect of constant temperatures and osmotic potentials on the germination of sugar beet. *Journal of Experimental Botany* 37: 729-741.

Hu XW, Fan Y, Baskin CC, Baskin JM and Wang YR. 2015. Comparison of the effects of temperature and water potential on seed germination of Fabaceae species from desert and subalpine grassland. *American Journal of Botany* 102: 649–660.

Walck JL, Hidayati SN, Dixon KW, Thompson K, Poschlod P. 2011. Climate change and plant regeneration from seed. *Global Change Biology* 17: 2145–2161.
